# PREVALENCE OF PORCINE CYSTICERCOSIS AMONG SCAVENGING PIGS IN WESTERN KENYA

**DOI:** 10.21010/ajid.v14i2.5

**Published:** 2020-07-31

**Authors:** Marie-Françoise Mwabonimana, Anthony Macharia King’ori, Charles Muleke Inyagwa, Eduard Kokan Shakala, Bockline Omedo Bebe

**Affiliations:** 1 Department of Animal Sciences, Faculty of Agriculture, Egerton University, P.O. Box 536-20115, Egerton, Kenya; 2 College of Animal Sciences and Veterinary Medicine, University of Rwanda, P.O. Box 210, Musanze, Rwanda; 3 Department of Veterinary Medicine, Faculty of Veterinary Medicine and Surgery, Egerton University, P.O. Box 536 – 20115, Egerton, Kenya

**Keywords:** Pig, *Taenia spps*, meat inspection, ELISA test, Slaughter slabs, Smallholder farmers

## Abstract

**Background:**

Porcine Cysticercosis (PC) infection is globally classified as a neglected and re-emerging tropical disease. The disease is endemic in Western Kenya yet smallholder farmers continue to practice scavenging pig production, thereby posing public health risk. This study determined the prevalence of PC infection at the farms and slaughter slabs in a cross-sectional survey in two Counties (Busia and Kakamega) of Western Kenya.

**Materials and Methods:**

Two hundred and eighty-seven (287) heparinized blood samples were collected at the farm from 162 households in 9 villages and 113 pigs from 5 slaughter slabs. The prevalence of PC was detected through meat inspection at slaughter slabs, and the prevalence of *Taenia solium* antigen determined by using the ApDia Ag-ELISA test at the farms and slaughter slabs.

**Results:**

At meat inspection, the PC prevalence was 1.8%, while prevalence of *Taenia Species* cysts detected with Ag-ELISA test was 3.8% at the farms, and 5.3 % at the slaughter slabs. The Ag-ELISA test had sensitivity of 100% (95% CI: 19.79– 100.00) and specificity of 96.4% (95% CI: 90.49– 98.84).

**Conclusion:**

The PC prevalence levels observed among scavenging pigs in Western Kenya should be a cause of public health risk concern. This observation warrant enforcing mandatory pig confinement, and use of latrines at the farms and meat inspection at local slaughter slabs. Further studies are recommended to identify different *Taenia* species in cysticercoids pigs in the region, which this study could not differentiate.

## Introduction

Porcine Cysticercosis (PC) is a zoonotic, neglected food-borne disease of global public health concern and trade implications. The disease manifests itself as seizures and death in pigs and humans (Wardrop *et al.*, 2016). Cysticercosis in pigs is transmitted by two species of tapeworms: *Taenia solium*, the zoonotic and *T. hydatigena*, the non-zoonotic with the latter being rare in Africa (Nguyen *et al.*, 2016; Gomez-Puerta *et al.*, 2019). The zoonotic tapeworm *T. solium* has a two host life cycles; the indirect cycle with humans as the definitive hosts, and in pigs as a normal intermediate host harboring the larval cysticerci (Donadeu *et al.*, 2017). Cysticercosis in pigs results from ingesting *T. solium* eggs directly by fecal-oral route, or from environments contaminated with human harboring adult *T. solium* (Fleury *et al.*, 2013). Human transmission of *T. solium* is typically through consumption of under-cooked pork or water containing fecal matter (indirect) (Kungu *et al.*, 2017). The World Health Organization (WHO) classify PC as a neglected and re-emerging tropical disease (WHO, 2017). The prevalence of PC in endemic African counties is variable, and up to 40% prevalence has been observed (Shonyela *et al*. 2018).

Prevalence of PC can vary with the diagnostic methods used (Shonyela *et al.*, 2018). For instance, prevalence estimated through tongue inspection is lower (9.4%) relative to prevalence estimated through postmortem examination (15%), while Enzyme-linked Immuno electro transfer Blot (EITB) technique and ELISA B158/B60 diagnosis is much higher (24.7 to 29.7%). This difference is due to the test sensitivity and specificity or the probability of having a positive or negative result. This emphasizes the necessity to complement screening (meat inspection) with diagnostic confirmatory tests (Ag-ELISA) that have high sensitivity and specificity in PC detection. This study was undertaken to determine the prevalence of PC at the farm level and slaughter slabs in Busia and Kakamega Counties of Western Kenya, a region with prominent scavenging pig production systems in the country and considered endemic region for PC.

## Materials and Methods

### Area of study

The study was conducted in Busia and Kakamega Counties of Western Kenya. These Counties were purposely selected because they have high scavenging pig population. The current pig population is unknown in the two Counties, but the last census indicated that Busia has 21,315 while Kakamega has 6,198 pigs (KNBS, 2009).

### Sample size determination

The pig sample size, determined by using a formula by Yamane (1967) was 400:


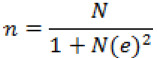


Where:

n is the corrected sample size needed; N is the pig population size, and e is 0.05 is the allowable margin of error set at 0.05.

### Procedure

A cross-sectional survey was conducted in nine villages in Busia and Kakamega Counties which were identified by the local veterinary office as having the highest numbers of pig-producing farmers and pig slaughter slabs. At farm level, pig producing households were randomly selected using simple random sampling procedure.

### Meat inspection

The meat inspection as screening test was performed at 5 local slaughter slabs in the two study Counties following the standard procedure (Harenda *et al.*, 2000). Results of day-to-day meat inspection were recorded for the pigs slaughtered during the study period.

### Blood sample collection and laboratory analysis

Five milliliters of whole blood were collected using vacutainer 10 ml syringes from the external ear vein or the jugular vein of pigs and stored in a cool box at 4 - 8 °C to prevent hemolysis during transportation to the laboratories. Samples from Busia Country were transported to the International Livestock Research Institute (ILRI) laboratory in Busia, while samples from Kakamega County were transported to the Veterinary Investigation Laboratory (VIL) in Kakamega. In both laboratories, blood samples were sedimented by centrifugation at 3000 rpm for 5 minutes at 20°C to obtain cleaner sera from pigs at farm and slaughter slabs levels in which circulating antigen were detected for viable parasites diagnosis at Egerton University. The serum was then dispensed into 2 milliliter labeled Eppendorf tubes and stored at 4 °C. The serum samples were submitted to molecular laboratory in Animal Science Department at Egerton University, Njoro, Kenya, for freezing and preservation in a cool box at 4 - 8 °C for later Antigen (Ag) ELISA tests. The serum samples were analyzed using the ApDia Cysticercosis Antigen (Ag) ELISA test (REF 650501), an Enzyme Immunoassay for the qualitative determination of viable cysts of *Taenia* *spp*. (Deckers *et al.*, 2010).

### Ethical consideration

This research was authorized for implementation by the National Commission for Sciences, Technology and Innovation (NACOSTI), Permit No: NACOSTI/P/19/80633/27786. At the county level, consent was obtained from the County Director of Veterinary Services (CDVS) and community leaders and participants in the study area.

### Data analysis

All observed data were recorded, entered in Microsoft Excel (2007) and exported to the Statistical Analysis System 9.2 (SAS, 2008). Results on positive PC cases from visual meat inspection and from antigen ELISA test, were analyzed for epidemiological measures using FREQ procedure of SAS (2008). The sensitivity and specificity of the diagnosis tests were computed from a two-by-two contingency table.

The prevalence was computed from the serological tests and ELISA positive cases at the farm and slaughter slab sampling, using the formula of Pfeiffer (2013):

Op= TD^+^/n x 100

Where:

Op = Observed Prevalence of disease, TD*^+^* = Total diseased positive pigs, n = Total pigs sample.

The sensitivity was computed as: Sen= (TP/(TP+FN)) where, Sen= sensitivity, TP= True Positive, and FN= False Negative.

The specificity was then calculated as: Spec = (TN/(TN+FP)) where, Spec = Specificity, TN= True Negative and FP=False Positive.

Bayer’s theorem was applied to compute the likelihood that carcasses testing positive indeed had PC infection. The probability of a pig having the disease at the slaughter slabs level, given a positive result was then calculated as: P(A/X) = P(X/A) *P(A)/(P(X/A) *P(A)+ (P(X/~A) *P(~A).

Where:

P(A) = The probability of having pigs with *Taenia* cyst; P(X/A) = The probability of having true positives pigs and (P(X/~A) = The probability of having false positives pigs.

The priori prevalence of PC in Western Kenya was assumed to be 5.3% (Thomas, 2013).

## Results

### Sample pig herd characteristics

Out of the 400-pig sample population, serum samples collected were 287 at the farms and 113 at the slaughter slabs. Of the slaughtered pigs, 74.3% (84/113) were in Busia and 25.7% (29/113) in Kakamega from a total of 162 households. A larger number of the slaughtered pigs were boars (66.7%) but at the farms, gilts (65.2%) were more than the boars (34.8%). Most of the sampled pigs (61.1%) were reared under the scavenging system, while some (38.9 %) were tethered during the day and released in the night.

### Prevalence of porcine cysticercosis

Table 1 shows that the prevalence of PC detected with Ag-ELISA test was 3.8% (95% CI 1.61 – 6.05%) at the farm and 5.3% (95% CI 1.18 – 9.44) at the slaughter slabs. The PC prevalence detected using Ag-ELISA was 2.1 to 2.9 times higher than that detected from meat inspections (1.8%: 95% CI 0.66 – 4.20).

**Table 1:** The PC prevalence of Ag-ELISA and meat inspection at farm and slaughter slabs levels

Sampling level	Tests	Number of Pigs	Test results	

			Positive (%)	Negative (%)
Farms	Ag-ELISA	287	3.8	96.2
Slaughter slabs	Meat inspection	113	1.8	98.2
	Ag-ELISA	113	5.3	94.7

### Sensitivity and specificity of the Ag-ELISA test

**Figure 1:**
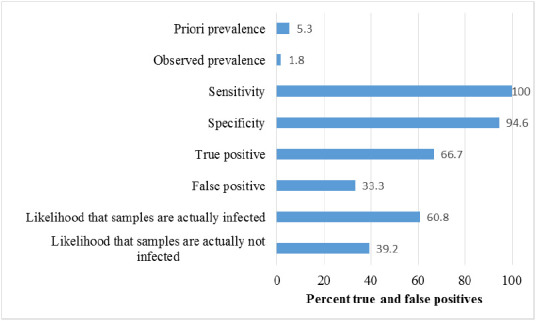
Percent True and False Positives for porcine cysticercosis

The Ag-ELISA test had sensitivity of 100% (95% CI: 19.79– 100.00) and specificity of 96.4% (95% CI: 90.49 – 98.84). The reliability of Ag-ELISA test was expressed by the true positive (66.7%, 95% CI: 5.999 – 75.8921) and false positive (39.2%, 95% CI: 24.1079 – 94.001) (Figure 1).

## Discussion

This paper reports the prevalence of Porcine Cysticercosis determined from a cross-sectional survey of pigs managed under scavenging system and subsistence livelihood in two Counties in Western Kenya. The scavenging system present high risk of PC infection and is in some countries a banned system of pig production. The observed PC prevalence from Ag-ELISA test at the farms (3.8%); at the slaughter slabs (5.3%) and County (6.9% in Kakamega and 4.8% in Busia) was much lower than the levels observed within other Counties in Kenya and in endemic African countries. Eshitera *et al*. (2012) estimated 32.8% PC prevalence in Homa bay County, but this county has a lower pig population compared to Busia and Kakamega (KNBS 2009). The PC prevalence in Homabay County is within the range of 19.5 to 40% reported in other East African countries as well as other African countries such as Ghana, Cameroun, Egypt and Mozambique (Shonyela et al. 2018) and South East Asia (Khaing *et al.*, 2015). The mean PC prevalence detected on meat inspection were higher compared to those reported in India (0.3% - 0.88%) by Satyaprakash *et al.*, (2018) and Vaidya *et al*. (2018), but lower than those (4.4%) reported in Nairobi, Kenya and South Western Nigeria (Akoko *et al.*, 2019; Adesokan *et al.*, 2019). These results would suggest that public health risk of porcine cysticercosis was relatively low in western Kenya.

Although public health risk may be low in Western Kenya, Porcine Cysticercosis is classified an emerging disease and globally targeted for total eradication, especially where pigs are produced under scavenging systems as it is the case in rural villages of Western Kenya. In Brazil, similar PC prevalence levels (5.3%) as those observed in Western Kenya was deemed high (Emilio *et al.*, 2017) in considering the global goal commitment to eradicate the disease by 2020. Considering that a large majority of the pigs were managed under scavenging conditions with low sanitary practices, public health risk of PC infections should be of concern in the region to warrant enforcing mandatory pig confinement, use of latrines at the farms, meat inspection at local slaughter slabs, and strengthened diagnostic surveillance. Implementation of risk-based surveillance at meat inspection would check against the potential for high economic losses, increased public health risks and pork trade in Western Kenya. This is important because prevalence of Porcine Cysticercosis infection in Western Kenya can be traced to contaminated environment and presence of a tapeworm carrier at the production level (Thomas, 2013; Shonyela *et al.*, 2018).

The observed PC prevalence in Western Kenya is within prevalence range (4.5 to 6.2%) observed in the same region between 2005 and 2013 (Thomas, 2013). This may be moderate prevalence compared to those exceeding 10% reported by WHO/FAO/OIE (2005) as highly endemic. Therefore, these results would suggest the presence of an endemic *Taenia species* infection in Western Kenya, probably sustained with predominant scavenging pig production under poor sanitary practices characterized by poor human fecal waste disposal. Use of latrines was limited in the area (27.2%). The poor pig management practices and inadequate meat inspection procedures sustains the life-cycle of *Taenia spps*., consequently sustaining endemic conditions and prevalence in both humans and pigs.

However, the present study could not differentiate between infections of different *Taenia* species in cysticercoids pigs as Ag-ELISA is genus specific and not species specific (*T.solium, T. hydatigena, T. asiatica*) (Devleesschauwer *et al.*, 2014). Further studies could pursue identification of different *Taenia* species in cysticercoids pigs in the region to better inform targeted interventions.

The sensitivity in this study was higher (100%) than 87% reported elsewhere (Krecek *et al.*, 2011). With the observed prevalence (1.8%), specificity (96.4% %), sensitivity (100%), true and false positives (33.3%, 66.7% respectively), carcasses that tested positive had 60.8% chance of being truly PC infected. Conversely, the probability that samples testing positive being actually not PC infected was 39.2%.

These results confirm findings by Fredriksson-Ahomaa, (2014) who stipulated that meat inspection should not be used alone because it leads to the underestimation of the disease prevalence in an endemic area. This, therefore, suggests that Ag-ELISA test should be used as a screening test, followed by meat inspection to confirm the infection at slaughter slabs.

## Conclusion and recommendation

Although it may be concluded that PC prevalence is relatively low in the Counties studied, public health risk intervention is warranted, considering that PC is a globally-neglected and re-emerging tropical disease. The intervention should involve enforcing mandatory pig confinement, use of latrines in the farms, meat inspection at local slaughter slabs and performing Ag-ELISA test of the parasite. Further studies are needed to identify the species of *Taenia* responsible for PC.

**List of Abbreviations:**
Ag: : Antigen
DVOs: : Directorate of Veterinary Services
ELISA: : Enzyme-linked immunosorbent assay
CESAAM: : African Centre of Excellence in Sustainable Agriculture and Agribusiness Management
EITB: : Enzyme-linked Immuno electro transfer Blot
ILRI: : International Livestock Research Institute
KNBS: :Kenya National Bureau of Statistics
NACOSTI: : National Commission for Sciences, Technology and Innovation
PC: : Porcine Cysticercosis
SAS: : Statistical Analysis System
*spp.*: : Species
*T. solium*: : *Taenia solium*
VIL: : Veterinary Investigation Laboratory
WHO: : World Health Organization.
